# Feasibility of a Community-Based, Online, Peer-Supported Spinal Cord Injury Self-management Intervention: Protocol for a Pilot Wait-Listed Randomized Trial

**DOI:** 10.2196/42688

**Published:** 2023-02-07

**Authors:** Susan Dunreath Newman, Sherwood Toatley, Marka Danielle Rodgers, Suparna Qanungo, Martina Mueller, Brian Denny, Angela Rodriguez

**Affiliations:** 1 College of Nursing Medical University of South Carolina Charleston, SC United States; 2 South Carolina Spinal Cord Injury Association Columbia, SC United States; 3 Center for Spinal Cord Injury Roper Rehabilitation Hospital Charleston, SC United States

**Keywords:** spinal cord injury, peer support, self-management, telehealth, community engaged research, community participation

## Abstract

**Background:**

People with spinal cord injury (SCI) report feeling unprepared to manage their disability upon discharge to the community. This situation is exacerbated when they return to settings where self-management support and resources are sparse, thus increasing the risk of costly secondary conditions and rehospitalizations. These factors make a compelling case for implementing innovative community-based SCI self-management programs that empower and engage individuals with SCI. Using a community-engaged research (CEnR) approach, we developed a peer-supported SCI self-management intervention, known as PHOENIX (Peer-supported Health Outreach, Education, and Information Exchange), which integrates online educational content and support from peer navigators (PNs) through telehealth, to promote health and community participation after SCI.

**Objective:**

The objective of this pilot study is to evaluate the feasibility and acceptability of PHOENIX and the study design, and to obtain estimates of the variability of relevant outcome measures.

**Methods:**

We conducted a pilot randomized waitlist-controlled trial (n=30) in collaboration with the South Carolina Spinal Cord Injury Association (SCSCIA), our long-standing community-based nonprofit organization research partner. We recruited 4 PNs through our SCSCIA collaboration using its existing network of trained peer mentors. Our study design supported comparison of the following 2 randomly assigned groups: PHOENIX intervention group and waitlist enhanced usual care (EUC) group. The PHOENIX intervention was administered online by PNs over 16 weeks through scheduled “video visits.” The EUC group participated in the study for 16 weeks with usual community services and no navigation, and received 4 monthly newsletters from the SCSCIA on a variety of SCI-relevant topics. At the end of the waitlist period, the waitlist EUC group received the full PHOENIX intervention. Measures of feasibility included PN and participant recruitment and retention, PN workload, protocol adherence, and incidence of technical issues. We conducted qualitative interviews with participants and PNs to evaluate the acceptability of PHOENIX and the study design. Outcome measures, including community participation, quality of life, and the occurrence and subjective impact of medically serious secondary conditions and rehospitalizations, were assessed at baseline after randomization and at subsequent time points to allow between-group comparisons.

**Results:**

PN hiring and training were completed in August 2018. Recruitment began in November 2018. A total of 30 participants were recruited across South Carolina, and 28 participants completed follow-up by August 2020. An analysis of the results is being finalized, and the results are expected to be published in 2023.

**Conclusions:**

This study will provide valuable information to guide future research seeking to address unmet self-management needs and improve outcomes in individuals with SCI. Feasibility findings of this study will provide evidence from CEnR guided by people with SCI and SCI service providers to inform further development, testing, and dissemination of effective and scalable self-management strategies for people with SCI.

**International Registered Report Identifier (IRRID):**

RR1-10.2196/42688

## Introduction

### Background

Spinal cord injury (SCI) is a life-altering event that results in varying degrees of paralysis, depending on the level and completeness of injury. According to the National Spinal Cord Injury Statistical Center, there are about 17,900 new SCI cases each year and about 296,000 people with SCI in the United States [1]. Presently, 87% of all people with SCI return to private noninstitutional residences in the community [2]. Individuals with SCI are among the most complex and costly patients to manage in the health care system, with approximately 30% of people with SCI being rehospitalized one or more times during any given year following injury. While the prevalence of SCI is relatively low in comparison with the prevalence of other chronic conditions, the costs are staggering. Estimates suggest that first-year postinjury health care and living expense costs average US $567,000 for individuals with paraplegia and US $1.16 million for quadriplegia, with estimated lifetime costs of up to US $5.16 million [1]. The treatment of secondary complications, such as pressure injuries and urinary tract infections, requiring rehospitalization further increases the cost.

Self-management is both a goal and a lifetime task after a traumatic disabling event such as SCI [3]. Individuals with SCI must engage in proactive self-care (eg, pressure relief, skin checks, and bladder and bowel care) to reduce the occurrence and severity of costly potentially preventable secondary conditions that can detract from community participation and reduce quality of life (QOL). Medical rehabilitation is an essential component of recovery and adaptation to life with SCI. However, the median rehabilitation length of stay has declined over the last 40 years from 98 days in the 1970s to 30 days currently [1]. Unfortunately, shorter rehabilitation results in reduced opportunities, before discharge to home, for education and practice in self-management skills that are essential to prevent common, albeit serious, postinjury secondary conditions. Rehospitalization and medically serious secondary conditions create risks for increased isolation, and decreased community participation and QOL [4,5].

People with SCI report feeling unprepared to manage their disability and its effects in an environment outside of the clinical setting [6]. This situation is exacerbated when they return to settings where self-management support and resources are sparse, thus increasing the risk of costly secondary conditions and rehospitalizations [7,8]. These factors make a compelling case for implementing innovative community-based SCI self-management programs that empower and engage individuals with SCI.

### Intervention Overview

Our strategy to promote SCI self-management has consistently involved specially trained peer navigators (PNs). In the context of our studies, PNs are people with SCI who are informed about their condition, take an active role in their self-care, are trained in key self-management components, and have a desire to help others learn to navigate life with a new disability [9,10]. As our pilot work and other studies demonstrate, learning from peers is vital in rehabilitation from a disabling injury and adjustment to living with SCI [11-14]. Our peer navigation model was initially pilot tested as an in-person intervention delivered in participants’ homes. However, given the issues of scalability and sustainability, scattered regional distribution of participants, access problems, and travel time and costs, we determined that a telehealth innovation was needed.

Our online PHOENIX (Peer-supported Health Outreach, Education, and Information Exchange) intervention evolved from lessons learned while piloting our in-person intervention. The primary goals of PHOENIX are to improve participants’ community participation and QOL, and the secondary goals are to decrease the occurrence and subjective impact of medically serious secondary conditions and rehospitalizations. The 16-week PHOENIX intervention consists of 3 key components: (1) education on post-SCI self-care, and community resource navigation and use; (2) skill optimization through role modeling by the PN, tailored participant-centered behavioral goal setting, and action planning; and (3) information exchange through shared problem solving and decision support with the PN. The primary modalities to support the telehealth delivery of PHOENIX include (1) web-based multimedia educational content and (2) scheduled televideo interactions, using a telehealth platform, for knowledge and skill building and information exchange between participants and PNs with SCI.

The purpose of this study was to pilot test, in collaboration with our long-standing community partner, the South Carolina Spinal Cord Injury Association (SCSCIA), a structured, sustainable, technology-enhanced SCI PN intervention (ie, “PHOENIX”) for statewide implementation across South Carolina. In congruence with the purpose of feasibility studies, the objective of this study was not to test the efficacy of the PHOENIX intervention but to determine whether the novel telehealth intervention and the study design could be feasibly implemented, and to obtain estimates of the variability of relevant outcome measures [15]. We designed a randomized pilot trial, using a waitlisted control group, to identify the potential logistical and methodological issues of both intervention implementation and study procedures in preparation for conducting a future full-scale randomized controlled trial [15]. We also sought to evaluate the PHOENIX intervention to obtain estimates of the variability of community participation and QOL (primary outcomes), and the occurrence and subjective impact of medically serious secondary conditions and rehospitalizations (secondary outcomes) to inform the design of an adequately powered future trial.

## Methods

### Community Engagement

Our approach reflected the Guidelines and Criteria for the Implementation of Community-based Health Promotion Programs for Individuals with Disabilities, including (1) use of an underlying theoretical framework; (2) implementation of process evaluation; (3) use of disability-appropriate outcome measures; (4) active involvement of people with disabilities in intervention development and implementation; (5) support of the personal beliefs, practices, and values of people with disabilities; (6) consideration of accessibility and barriers to program participation; and (7) sensitivity to financial constraints often experienced by people with disabilities [16]. A long-standing community-engaged research partnership between the principal investigator (SDN) and the SCSCIA supported this line of research. Over the course of our partnership, research activities have been guided by an active and empowered community advisory board comprised of community members with SCI, and representatives from agencies providing services to people with SCI. The advisory board participated in the conceptualization of PHOENIX and the development of multimedia content for the intervention to assure relevance and accessibility of the material. They also provided feedback on the structuring and layout of the online educational curriculum. The SCSCIA collaborated with the study team on (1) expansion and formalization of the PN training curriculum; (2) identification and recruitment of individuals who would serve well as PNs; (3) facilitation of PN training workshops; (4) periodic monitoring of PN performance; (5) assistance with participant recruitment; and (6) support to the principal investigator in addressing additional navigator training needs over the course of the study. It is also engaged in assisting with the dissemination of study results to nonacademic audiences in the community.

### Study Design

**Figure 1:**
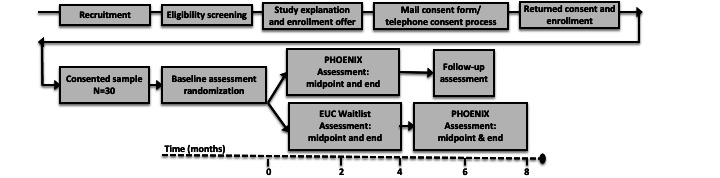
Study design and flow. EUC: enhanced usual care; PHOENIX: Peer-supported Health Outreach, Education, and Information Exchange.

Advisory board guidance was sought to address scientific rigor and logistics, as well as ethical concerns about condition assignment. We addressed these concerns through a randomized waitlist study design, so all participants received the intervention. Our study design supported the conduct of a pilot randomized (individual level) waitlist-controlled trial with 30 adults with SCI, comparing 2 groups: a waitlist enhanced usual care (EUC) group and a PHOENIX intervention group. At the end of the waitlist period, the EUC group received the full intervention (Figure 1).

### PN Recruitment, Training, and Retention

We recruited 4 PNs through our SCSCIA collaboration using its existing network of trained peer mentors. The PNs completed 2 full days of training on implementation of the PHOENIX intervention and working on a research team, which included completion of training in protecting human subjects, research ethics and compliance, and maintaining participant privacy and study data confidentiality. An additional full-day training was provided approximately 6 months after study initiation. To promote retention, all PNs were hired and paid as university employees. Additional retention strategies included scheduled weekly meetings with the team over the course of the study to address issues that arose in the navigation process and to provide ongoing support to PNs.

### Ethics Approval

Institutional Review Board (IRB) approval (Medical University of South Carolina; number: Pro00071526) was obtained prior to the initiation of study participant recruitment. As the SCSCIA was determined to be engaged in research, a certificate of Federal Wide Assurance was obtained with a reliance agreement in place with the university IRB.

### Inclusion and Exclusion Criteria

The inclusion criteria were as follows: (1) South Carolina resident; (2) ≥18 years old; (3) chronic paralysis due to traumatic SCI; (4) level and severity of paralysis requiring locomotion with a wheelchair for >6 hours/day; (5) living in a private residence; (6) accessible by mail, phone, or email; and (7) able to speak English. The exclusion criteria were as follows: (1) cognitive or mental impairment that would prevent informed consent; (2) level of injury requiring use of a ventilator; (3) unhealed stage III or IV pressure ulcer requiring bed rest; (4) acute serious medical illness at the time of screening that would inhibit participation in the intervention; and (5) plans to move out of South Carolina in the next 8 months.

### Number of Subjects

Our targeted sample was 30 adults with SCI from urban and rural regions across South Carolina. Consistent with guidance provided by Leon et al and Tickle-Degnen, our intent with this pilot study was not to estimate effect size, and our sample size was determined primarily by pragmatic reasons [15,17,18]. Sample size determination was based on estimates of workload for PNs. We expected that PNs could work with 2 study participants at a time during the first 8 weeks of the intervention that required weekly contact. For a sample size of 15 participants per group (intervention and waitlist control), the between-group difference in change scores for the relevant outcome measures for the intervention group versus waitlist control group was estimated with precision to range from ±7.6 to ±15.1 for SDs of difference scores ranging from 15.0 to 30.0 SD units.

### Recruitment and Consent

Established collaborations, including the SCSCIA’s network of peer support groups and relationships with SCI-related service providers and rehabilitation hospitals, supported recruitment of 30 adults with SCI from urban and rural regions across South Carolina. We announced the study through flyers, SCSCIA staff word of mouth, the SCSCIA Facebook page and other social media, and the SCSCIA website. Study announcements provided study team contact information to individuals interested in participating.

Once an individual responded to the call for participants, we conducted screening procedures to determine eligibility, thus following CONSORT (Consolidated Standards of Reporting Trials) guidelines to determine detailed information on recruitment flow. Eligible individuals were then provided with a brief study description, including randomization, and a general schedule of procedures to assess continued interest prior to conducting the informed consent process.

We offered 2 options for completing the informed consent process, by telephone or eConsent through REDCap (Research Electronic Data Capture). For telephone consent, eligible and interested individuals provided their full name and contact information. We then mailed 2 copies of the informed consent to the interested individuals and scheduled a time to complete the consenting process by phone, after which the participants signed and returned 1 signed copy of the consent. For eConsent, individuals interested in study participation provided their full name and contact information, including an email address, to which we sent a REDCap survey link containing the informed consent document. Study staff scheduled a time to complete the consenting process by phone, after which the participants signed the eConsent in REDCap. Upon submitting the eConsent, a REDCap trigger immediately notified the person obtaining consent, who then signed the document. Participants could download a copy of their executed informed consent directly to their own computer or request a copy be emailed or mailed to them.

### Randomization

Once signed consent was received, participants were randomized and placed in the appropriate group in the REDCap database. Randomization was at the participant level, using the REDCap automated randomization feature. Following the completion of baseline measures, staff informed participants of their group assignment.

### Intervention Delivery Platform

#### iTunes U

The iTunes U environment provided a private 1-way (ie, study participants did not enter any personal information into iTunes U) platform for the dissemination of multimedia educational content, including web links, documents, and videos, addressing topics in the PHOENIX curriculum (the educational videos are available online [19]). The educational content served as a source of information, as well as provided topics/themes for PN and participant discussions during the video visits. The educational content in iTunes U provided a library of resources available to PNs and participants, which was used to tailor the information provided, and an approach to address the relative learning needs of each participant.

#### Doxy.me

The low bandwidth televideo solution Doxy.me was used to facilitate the video visits between PNs and participants. Doxy.me is a Health Insurance Portability and Accountability Act–compliant, free, and secure telehealth solution that does not require downloads, plugins, or specialized hardware, and works natively on iPads through the Doxy.me app.

#### iPads

Once participants were assigned to PHOENIX, either at randomization or the end of waitlist, we assessed assistive technology needs, and participants were sent a kit by certified mail, which contained an iPad preloaded with PHOENIX educational materials, any required assistive technologies, and instructions on operation of the iPad. After we received confirmation that the iPad was received, study staff placed an initial call to assist iPad set up and provide information on the assigned PN who contacted the participant and initiated PHOENIX. The iPad provided a means for participants to access educational materials in iTunes U and engage in video visits using the Doxy.me app with PNs. In return for participants’ time and effort for participating in the study, the study team transferred ownership of the iPad to the study participants, and they retained any additional assistive technologies provided to them at the end of the study. At the end of the study, the cellular data plan provided by the study was terminated, and participants were provided with suggestions for free Wi-Fi access in their community (eg, public library).

### Intervention Implementation

PHOENIX was administered by the PN over 16 weeks. The first 8 weeks consisted of 6 educational modules (Table 1) and weekly televideo contact (ie, “video visits”) via Doxy.me with the PN, followed by (weeks 9-16) biweekly scheduled video visits with the PN to address progress to goals; barriers encountered; skin, bladder, or bowel issues; or hospitalization since last contact. PHOENIX integrated the key skills of self-management as defined by Lorig et al: problem solving, decision making, resource utilization, formation of partnerships, action planning, and self-tailoring [3]. Developing participant self-efficacy in these essential skills through construction of action plans and facilitated goal setting supported a participant-centered approach to self-management. PNs facilitated and modeled methods to access community services and resources, as well as incorporated aspects of peer mentoring by helping the individual establish goals, served as a resource for advice and guidance, and modeled strategies to meet goals [20]. The intervention was designed to promote participant efficacy in achieving a desirable level of community participation and QOL through knowledge and skill building related to self-management, guided by motivation and support from PNs.

**Table 1:** PHOENIX^a^ curriculum.

Week	Content/activity	PN role^b^
Week 1: Introduction to PHOENIX and SCI^c^ 101	What is PHOENIX? Relationship building exercise Brief PN video bios Understanding your SCI Initial personal goal setting	Describe role of the PN Share personal story Engage peer in story sharing Use action planning strategies to identify goals Facilitate realistic goal setting and identifying potential barriers
Week 2: Getting what you need: Being an empowered consumer	Self-advocacy skills Knowing your rights Active versus passive communication	Role play Facilitate discussion of a video Assess progress to personal goal (PTPG) Facilitate problem solving barriers
Week 3: Getting out there: Engaging community resources	Identifying resources to support personal goal attainment Initiate contact with a resource	Assist with locating relevant resources Support peer in engaging a resource Assess PTPG Facilitate problem solving barriers
Week 4: Staying healthy: Skin care and preventing pressure injury	Skin care after SCI Pressure injury prevention Identifying a problem and taking action	Evaluate knowledge/address gaps Share personal experiences and strategies Assess PTPG Facilitate problem solving barriers
Week 5: Staying healthy: Preventing urinary tract infection (UTI)	Bladder management after SCI UTI prevention Identifying a problem and taking action	Evaluate knowledge/address gaps Share personal experiences and strategies Assess PTPG Facilitate problem solving barriers
Week 6: Staying healthy: Bowel management	Bowel management after SCI Identifying a problem and taking action	Evaluate knowledge/address gaps Share personal experiences and strategies Assess PTPG Facilitate problem solving barriers
Weeks 7, 8, 10, 12, 14, and 16: Follow-up	Video visit for action planning and goal support	Share personal experiences and strategies Assess PTPG Facilitate problem solving barriers

^a^PHOENIX: Peer-supported Health Outreach, Education, and Information Exchange.

^b^PN: peer navigator.

^c^SCI: spinal cord injury.

### Comparison Condition

The EUC waitlist group participated in the study for 16 weeks with usual community services and no navigation. Waitlist participants received 4 monthly newsletters from the SCSCIA on a variety of SCI-relevant topics. The newsletters contained contact information should participants need information or assistance. Following the end of waitlist assessment, waitlist participants crossed over to the PHOENIX intervention group. To compensate the waitlist participants for the completion of additional measures before crossover to PHOENIX and receiving the iPad, we provided US $50 payment for completing baseline and week 8 study measures.

### Data Collection and Analysis: Feasibility

Assessment of feasibility was guided by Leon et al and Tickle-Degnen [15,18]. We assessed the feasibility of both the intervention and the study design and research procedures. Specific intervention and study components that were assessed, and their relevant quantifications are mentioned in Table 2.

**Table 2:** Feasibility components and quantification.

Component		Feasibility quantification
**Intervention component**		
	Navigator workload	Time logs (minutes per activity)
	Retention	PN^a^ attrition rate
	Fidelity	Rates of adherence to intervention protocol by PNs
	Adherence	Rates of adherence to intervention protocol by participants
	Online module access (dosage)	Use statistics – number and duration of educational module access
	Televideo access (dosage)	Use statistics – number and duration of televideo meetings
	Technology	Rates of device and connectivity issues
	Safety	Rate of adverse events
**Study component**		
	Screening	Number screened per month
	Recruitment	Number enrolled per month
	Enrollment and consenting	Duration of enrollment and consenting process
	Randomization	Proportion of screen eligible individuals who enrolled
	Retention	Intervention versus waitlist attrition rates
	Outcome assessments	Proportion of planned assessments completed; time to complete measures

^a^PN: peer navigator.

In addition to standard measures of feasibility, expanding the reach of PHOENIX across South Carolina using a telehealth approach removed the in-person procedures used in the original PN pilot, thus creating the need for study procedures that could be completed at a distance. We also evaluated the feasibility and logistics of our proposed screening, enrollment, and consenting procedures for use in a future larger trial with broader reach.

Part of our feasibility evaluation included determining the time required by PNs to complete navigation tasks through a telehealth platform. We closely monitored navigator workload and effectiveness. To support PN retention and closely monitor the PN experience, we held weekly team meetings, including the investigators, SCSCIA executive director, SCSCIA community outreach coordinator, and PNs, to assess and discuss issues uncovered by PNs in interactions with study participants and to provide support and guidance for addressing these issues. The proceedings of these meetings were audio recorded for later analysis and use in quality and process evaluation.

### Intervention Fidelity

Standardized training of PNs and use of intervention manuals that listed session objectives and format, instructional resources, session content, and monitoring criteria supported fidelity in intervention delivery. Tracking logs in REDCap were completed by PNs at each study visit (Multimedia Appendix 1) and were used to monitor the dosage/fidelity of PNs’ contacts, including frequency, length of contact, predominant interaction content, and participant progress. Feasibility will be evaluated by reporting 95% CIs on proportions and differences in proportions between the 2 groups for categorical feasibility measures, such as recruitment, retention, and adherence, and via 95% CIs for means and differences in means for continuous feasibility outcomes, including number of modules accessed, number of video visits completed, duration of video visits, etc.

### Acceptability

We conducted postintervention evaluation surveys (Table 3) and interviews (Textbox 1) with participants, and a summative debriefing session with PNs to assess the usefulness, acceptance, and satisfaction regarding the waitlist study design and intervention components, as well as participant response burden for completing measures. Participants were asked about feedback regarding their experiences with engaging PNs, the use of the iPads and any other technology, the presence of any safety or comfort-related concerns about the study, and problems with the navigation process. The interviews and debriefing session were audio recorded, transcribed, and analyzed for process evaluation purposes, using the methods of qualitative content analysis.

**Table 3:** Postintervention evaluation survey items.

Item		Response options
**Please select the answer that best describes your feeling about each statement**		
	The length of partnership with my peer navigator was…	Too short, too long, or just right
	The number of times I met with the peer navigator was…	Not enough, too often, or just right
	How satisfied were you with the peer navigator?	Not, somewhat, or very
	How satisfied were you with the educational materials?	Not, somewhat, or very
	How satisfied were you with using iTunes U?	Not, somewhat, or very
	How satisfied were you with using the iPad?	Not, somewhat, or very
	How comfortable were you with video visits with the peer navigator?	Not, somewhat, or very
**Please indicate how much the PHOENIX^a^ program helped you in the following areas...**		
	My ability to speak up and advocate for my needs	No help, some help, or great deal of help
	My knowledge about community resources & services	No help, some help, or great deal of help
	My ability to access resources & services that I need	No help, some help, or great deal of help
	My ability to make decisions that affect my health	No help, some help, or great deal of help
	My ability to solve problems that arise	No help, some help, or great deal of help
	Building a support system	No help, some help, or great deal of help
	My ability to participate in activities that interest me	No help, some help, or great deal of help
	Feeling I’m in control over my life	No help, some help, or great deal of help
	My knowledge about spinal cord injury	No help, some help, or great deal of help
	My ability to prevent pressure ulcers	No help, some help, or great deal of help
	My ability to prevent urinary tract infections	No help, some help, or great deal of help
	My ability to prevent bowel problems	No help, some help, or great deal of help

^a^PHOENIX: Peer-supported Health Outreach, Education, and Information Exchange.

**Evaluation interview questions.**

**Questions**

- What were your expectations for the PHOENIX (Peer-supported Health Outreach, Education, and Information Exchange) program before you began?

- What are the most helpful parts of the PHOENIX program in general?

- What are the least helpful parts of the PHOENIX program in general?

Now let’s talk about using technology in PHOENIX.

First let’s discuss the educational materials that were provided through iTunes U.

- Did you find these materials helpful?

- What did you like about them?

- What would make them better?

- Was any information missing?

- Describe your experience in using the iPad to video chat with the peer navigator.

- Describe any technology problems or challenges you experienced.

- Did you use the iPad to access other SCI (spinal cord injury) information on the internet? If yes, tell me more about this.

Now let’s talk about your experience in working with the peer navigator.

- How would you describe the nature of your relationship with your peer navigator?

- What did you like about working with a peer navigator?

- What would make this experience better?

- Goal setting and following up on your goals with your peer navigator is an important part of the program. Tell me a little bit about your experiences with setting goals while you were in PHOENIX.

- Tell me about what you learned about self-advocacy while you were involved with this program.

- Describe any changes in your life with SCI that resulted from participating in PHOENIX.

- What suggestions do you have to make this program better?

- Do you have any other last thoughts that you would like to share?

### Data Collection and Analysis: Outcomes

Baseline data collection occurred after randomization (week 0). In the intervention group, we collected outcome measures at the intervention midpoint (week 8) and end (week 16), and at follow-up (week 24). In the waitlist control group, we collected outcome measures at the same time points to allow preliminary between-group comparisons, including at baseline (week 0), waitlist midpoint (week 8), end of waitlist/preintervention (week 16), intervention midpoint (week 24), and intervention end (week 32) (Figure 1). We used REDCap for data collection and management. Participants were provided with a link to access the REDCap survey using their iPads. Contact information was provided to allow the option of phone administration for participants who were not able to complete the REDCap survey.

### Measures

We used established instruments with reported validity and reliability in populations with SCI (measures and timing are detailed in Table 4). We used relevant item banks from the Spinal Cord Injury-Quality of Life (SCI-QOL) instrument developed for use with SCI populations, which builds on the Patient-Reported Outcomes Measurement Information System (PROMIS) and the Quality of Life in Neurological Disorders (Neuro-QOL) initiative [21]. We used short-form instrument options when available to minimize participant response burden.

**Table 4:** Measures and timing.

Domain			Measures		Timing
**Outcomes**					
	Community participation (objective)	Craig Handicap Assessment & Reporting Technique (CHART): Mobility subscale items [22,23]		All time points^a^	
	Community participation (subjective)	- Reintegration to Normal Living Index (RNL), 10 items [24-26] - SCI-QOL^b^ Ability to Participate (ATP), 10 items; and Satisfaction with Social Roles (SRS), 10 items [27]		All time points^a^	
	Quality of life	SCI-QOL Positive Affect & Wellbeing (PAWB), 10 items [28]		All time points^a^	
	Secondary conditions and rehospitalizations	- SCI-QOL Pressure Ulcers Scale (PUS), 7 items [29] - Bladder Complications Scale (BCS), 5 items [30] - Bowel Management Difficulties Scale (BMD), 8 items [30] - Self-report incidence of PU^c^, UTI^d^, and rehospitalization		All time points^a^	
**Mediators**					
	Self-efficacy	Moorong Self-Efficacy Scale (MSES), 16 items [31,32]		All time points^a^	
	Social support	Medical Outcomes Survey-Social Support Scale (MOS-SSS), 19 items [33]		All time points^a^	
	Knowledge	Secondary conditions quiz, 10 items		All time points^a^	
**Moderators**					
	Demographics	Self-report		Baseline	
	SCI^e^ information	Self-report		Baseline	
	Technology	Self-report		Baseline	
	Stigma	SCI-QOL Stigma, 10 items [34]		Baseline, end, and follow-up	
	Anxiety and depression	Hospital Anxiety and Depression Scale (HADS), 14 items [35]		Baseline, end, and follow-up	

^a^All time points include weeks 0, 8, 16, and 24 for both groups, and week 32 for the waitlist group.

^b^SCI-QOL: Spinal Cord Injury-Quality of Life.

^c^PU: pressure ulcer.

^d^UTI: urinary tract infection.

^e^SCI: spinal cord injury.

### Data Analyses

As this pilot study was intended to assess feasibility and was not a hypothesis testing study, no inferential statistical tests were proposed [15]. Descriptive statistics were calculated, as appropriate, for all variables for the total sample and within the PHOENIX and control groups. The primary purpose of this data analysis was to generate estimates of variability in the primary and secondary outcomes. The primary continuous outcome measures to be used to evaluate peer navigation efficacy in a future larger adequately powered trial are community participation (Craig Handicap Assessment & Reporting Technique [CHART], Reintegration to Normal Living Index [RNL], and SCI-QOL Ability to Participate [ATP] & Satisfaction with Social Roles [SRS]) and QOL (SCI-QOL Positive Affect & Wellbeing [PAWB]) scores, and the secondary outcome measures are occurrence and subjective impact of secondary conditions (SCI-QOL Pressure Ulcers Scale [PUS] & Bladder Complications Scale [BCS]) obtained at baseline and 2 and 4 months. In addition, within the PHOENIX group, the change from baseline to 6-month follow-up was assessed for community participation and QOL. We obtained 95% CIs for the unadjusted as well as adjusted mean change from pretreatment to posttreatment (baseline to 2 and 4 months) for primary and secondary outcome measures within each group and for differences in the change from pretreatment to posttreatment between the 2 groups (PHOENIX and EUC). Though CIs obtained as estimates of variability for the relevant outcome measures were wide, as expected from a feasibility study, they provided reasonable estimates as input for the determination of effect sizes in future trials. Given that estimates from small studies carry uncertainties for the calculation of sample size for future studies, we plan to carry out sensitivity analyses to assess deviations from variability assumptions, as suggested by Julious and Owen [36]. Future investigations will explore the effects of demographic (age, race/ethnicity, gender, education, income, and living situation) and clinical (injury level, injury severity, time since injury, stigma, anxiety, and depression) characteristics, as well as potential mediating (self-efficacy, social support, and patient activation) variables on community participation and QOL through inclusion as adjustment variables; thus, we piloted the collection of these variables in this study.

## Results

**Figure 2:**
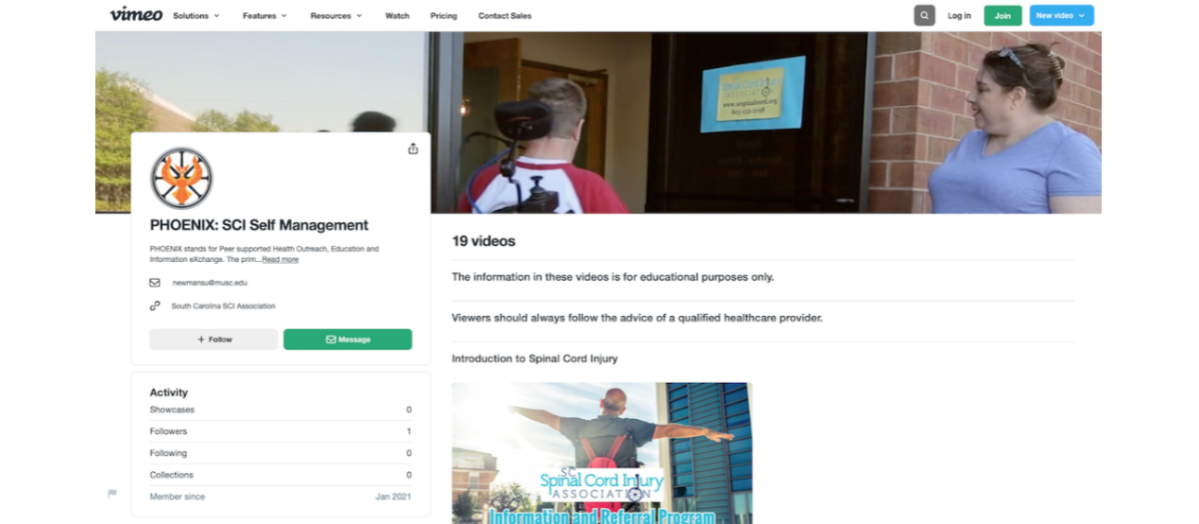
PHOENIX online educational content: Vimeo website landing page. PHOENIX: Peer-supported Health Outreach, Education, and Information Exchange.

**Figure 3:**
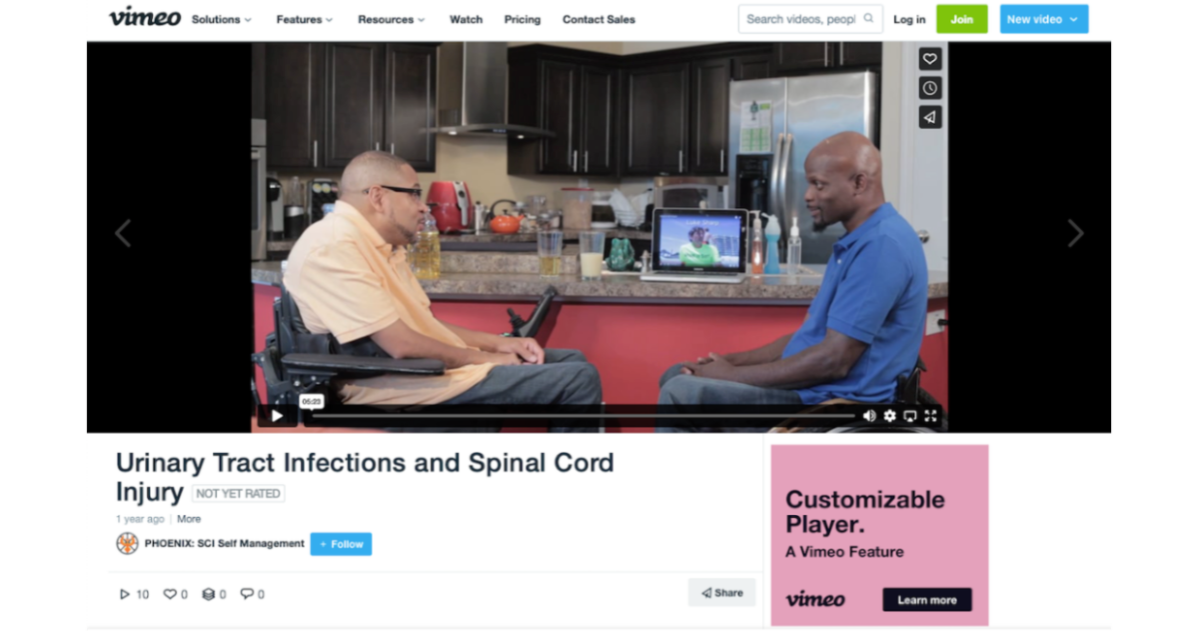
PHOENIX online educational content: Vimeo website educational video example. PHOENIX: Peer-supported Health Outreach, Education, and Information Exchange.

PN training was completed and IRB approval for the study was obtained in August 2018. The first participant was enrolled in early November 2018. Due to the pattern generated by the automated randomization feature in REDCap, 8 out of 12 participants in the first wave of the study were randomized to the active intervention, and 4 were randomized to the waitlist arm. By the end of November, our PNs had reached their maximum capacity of 2 participants each; thus, we paused enrollment and placed interested individuals on a study enrollment waitlist. As consented participants completed the active intervention, additional participants were consented and enrolled. The final participant postintervention interview was completed in August 2020, and a debriefing focus group with PNs was completed in November 2020. Initial analysis of feasibility and outcome data was completed in September 2021. The study results have been disseminated to community partners involved in the development of the PHOENIX intervention, and the multimedia educational content (Figures 2 and 3) is available on the SCSCIA website via Vimeo [19] since the discontinuation of the iTunes U platform by Apple in 2021. The findings will be submitted to peer-reviewed journals and presented at academic conferences [37]. An analysis of the results is being finalized, and the results are expected to be published in 2023.

## Discussion

### Principal Findings

Our research addresses an innovative combination of self-management and rehabilitation science, that is, the integration of peer-support, navigation, and telehealth to create an internet-based self-management intervention that is accessible, has extensive reach potential, and is designed to address the chronic consequences of an often permanently disabling injury with great individual and community burden. Use of a community-engaged research approach helps to ensure that the PHOENIX intervention is designed to be highly relevant to the unique needs of individuals with SCI, and that the study design and procedures are acceptable to the participant population. In congruence with the purpose of feasibility studies, the objective of this study is not to test for efficacy but to determine whether the PHOENIX intervention and the study design can be feasibly implemented, and to obtain estimates of the variability of relevant outcome measures. The field testing of the PHOENIX intervention through a randomized pilot trial using a waitlisted control group will facilitate the identification of potential logistical and methodological issues of both intervention implementation and study procedures. Specifically, our pilot trial is designed to evaluate aspects of feasibility and acceptability systematically, inform refinement of intervention components and delivery, and strengthen study procedures and processes required to conduct a robust future large-scale trial. Subsequent rigorous testing of the intervention will ultimately contribute to the scientific knowledge base regarding effective community-based self-management following a traumatic disabling injury, particularly those for which paralysis is central. This line of research will provide valuable information to support our long-term goal of developing an intervention that integrates online educational content and support from PNs through telehealth in order to promote effective self-management after SCI and ultimately increase the capacity of individuals with SCI to maintain health and participate in the community as desired.

### Possible Challenges, Limitations, and Solutions

Designing and implementing a community-based trial using participatory methods with a historically vulnerable population while maintaining scientific rigor are quite challenging. We will attempt to maintain internal validity and minimize selection bias through randomization and by inviting all potentially eligible participants to join the study. We will report the demographic characteristics of our study participants and will compare these with published characteristics of the target group. We did not identify a specific time since injury in our recruitment criteria, as we did not want to eliminate the possibility of recognizing potentially unique needs related to SCI of a long duration. Regarding external validity, we recognize that the results of this pilot study cannot be generalized to the entire population with SCI but may be representative of the target population of people with SCI residing in the study area to the extent that those who volunteer to participate in our study sample are representative of this target population. We will monitor issues related to intervention fidelity and feasibility that can inform strategies to minimize threats to internal and external validity.

### Conclusion

The results of this study will provide evidence from community-engaged research guided by people with SCI and SCI service providers to inform future development, refinement, testing, and dissemination of effective and scalable self-management strategies for people with SCI.

## Appendix

Multimedia Appendix 1 Example REDCap (Research Electronic Data Capture) form: Peer navigator documentation of study visits.

Multimedia Appendix 2 Peer-review report by the National Institute on Disability, Independent Living, and Rehabilitation Research (NIDILRR) - Field Initiated Projects Program (Research) Grant Competition (Washington, DC).

## Abbreviations

EUC: enhanced usual care
IRB: Institutional Review Board
PHOENIX: Peer-supported Health Outreach, Education, and Information Exchange
PN: peer navigator
QOL: quality of life
REDCap: Research Electronic Data Capture
SCI: spinal cord injury
SCI-QOL: Spinal Cord Injury-Quality of Life
SCSCIA: South Carolina Spinal Cord Injury Association

## Data Availability

The data sets analyzed during this study are available from the corresponding author on reasonable request.
