# Supplementary material for: Feasibility of a Community-Based, Online, Peer-Supported Spinal Cord Injury Self-management Intervention: Protocol for a Pilot Wait-Listed Randomized Trial
Source: JMIR Res Protoc. 2023 Feb 7;12:e42688. doi: 10.2196/42688 (PMC9944143; doi:10.2196/42688)
Supplement: Multimedia Appendix 1 [file resprot_v12i1e42688_app1.pdf]

Week 1 Navigator Session Checklist

Participant ID __________________________________

Week One: Introduction to PHOENIX & SCI 101

Week 1 session date: __________________________________

Review iTunes U content on PHOENIX:

☐ Review PHOENIX program info
☐ Identify goals of the program
☐ Define role of the Peer Navigator (what you can & cannot do)
☐ Provide your availability schedule

Review iTunes U content on SCI 101:

☐ Have participant describe his/her injury
☐ Review SCI 101 content as needed with participant to clarify his or her understanding of his/her injury

Relationship Building Exercise:

☐ Share your story
☐ Encourage the Peer to share his/her story

SMART Planning

Goal Setting: Is there anything you would like to do or accomplish in the next week or two?

◯ Yes
◯ No
◯ Not sure

After offering a couple ideas, has the participant identified any goals they would like to acheive?

◯ Yes
◯ No

What is it that you would like to specifically do or achieve?

On a scale of 0 to 10, where 10 is totally confidant and 0 is not confident at all, how confident or sure do you feel about carry out your plan?

- ◯ 0
- ◯ 1
- ◯ 2
- ◯ 3
- ◯ 4
- ◯ 5
- ◯ 6
- ◯ 7
- ◯ 8
- ◯ 9
- ◯ 10

Now, on the same scale of 0 to 10, how confident or sure do you now feel about carry out your plan?

- ◯ 0
- ◯ 1
- ◯ 2
- ◯ 3
- ◯ 4
- ◯ 5
- ◯ 6
- ◯ 7
- ◯ 8
- ◯ 9
- ◯ 10

Now, on the same scale of 0 to 10, how confident or sure do you now feel about carry out your plan?

- ◯ 0
- ◯ 1
- ◯ 2
- ◯ 3
- ◯ 4
- ◯ 5
- ◯ 6
- ◯ 7
- ◯ 8
- ◯ 9
- ◯ 10

Would it be helpful to set up a check on how things are going with your plan?

- ◯ Yes
- ◯ No

Would it be OK for us to discuss your progress to meeting your goal at our next visit?

◯ Yes
◯ No

Technology Issues

Did you experience any technological issues during the session?

◯ Yes
◯ No

Describe technology issues encountered:

Navigator Section

Duration of session in minutes: __________________________________

Date for next meeting: __________________________________

Time for next meeting: __________________________________

Peer Navigator

◯ Lamb
◯ Markatine
◯ Rodgers
◯ Toatley

Additional Comments/Notes

Week 2 Navigator Session Checklist

Participant ID	__________________________
Week 2 session date:	__________________________
Week Two: Getting What You Need: Being an Empowered Consumer	
Review iTunes U content on Self-Advocacy:	☐ Discussion of video scenarios
Progress to Peer Sharing:	☐ Peer describes poor service or discrimination experience<br>☐ Peer describes potential self-advocacy strategies to address this experience
Role play:	☐ Peer practices self-advocacy skills<br>☐ Provide constructive feedback
Planning:	☐ Anticipate need to use self-advocacy skills<br>☐ Solicit ideas regarding how they plan to approach this situation<br>☐ Provide constructive feedback on their plan

SMART PlanningPrevious week's plan: [week_17__pn_s1_arm_1][plan1_wk1] [week_1__pn_s1_arm_2][plan1_wk1]

Did the participant make a SMART plan last week?	◯ Yes<br>◯ No
What progress was made towards the plan?	◯ Completed<br>◯ Partially completed<br>◯ Did not carry out plan

Goal Setting: Is there anything you would like to do or accomplish in the next week or two?

◯ Yes
◯ No
◯ Not sure

After offering a couple ideas, has the participant identified any goals they would like to acheive?

◯ Yes
◯ No

What is it that you would like to specifically do or achieve?

On a scale of 0 to 10, where 10 is totally confidant and 0 is not confident at all, how confident or sure do you feel about carry out your plan?

◯ 0
◯ 1
◯ 2
◯ 3
◯ 4
◯ 5
◯ 6
◯ 7
◯ 8
◯ 9
◯ 10

Now, on the same scale of 0 to 10, how confident or sure do you now feel about carry out your plan?

◯ 0
◯ 1
◯ 2
◯ 3
◯ 4
◯ 5
◯ 6
◯ 7
◯ 8
◯ 9
◯ 10

Now, on the same scale of 0 to 10, how confident or sure do you now feel about carry out your plan?

◯ 0
◯ 1
◯ 2
◯ 3
◯ 4
◯ 5
◯ 6
◯ 7
◯ 8
◯ 9
◯ 10

Would it be helpful to set up a check on how things are going with your plan?

◯ Yes
◯ No

Would it be OK for us to discuss your progress to meeting your goal at our next visit?

◯ Yes
◯ No

Technology Issues

Did you experience any technological issues during the session?

◯ Yes
◯ No

Describe technology issues encountered:

Navigator Section

Duration of session in minutes: ______________________________

Date for next meeting: ______________________________

Time for next meeting: ______________________________

Peer Navigator

◯ Lamb
◯ Markatine
◯ Rodgers
◯ Toatley

Additional Comments/Notes
