# Supplementary material for: Feasibility of a Community-Based, Online, Peer-Supported Spinal Cord Injury Self-management Intervention: Protocol for a Pilot Wait-Listed Randomized Trial
Source: JMIR Res Protoc. 2023 Feb 7;12:e42688. doi: 10.2196/42688 (PMC9944143; doi:10.2196/42688)
Supplement: Multimedia Appendix 2 [file resprot_v12i1e42688_app2.pdf]

DEPARTMENT OF HEALTH & HUMAN SERVICES
Administration for Community Living
Washington, D.C. 20201

September 8, 2017

Susan Newman, CRRN, PhD, RN
99 Jonathan Lucas Street
MSC 160
Charleston, SC 29425

Applicant Name: ***Medical University of South Carolina***
Application Number: ***IFRE17000009***

Dear Dr. Newman:

Congratulations! Your application to the National Institute on Disability, Independent Living, and Rehabilitation Research (NIDILRR) for consideration under the Fiscal Year 2017 ***Field Initiated Projects Program (Research) Grant Competition (IFRE-0192)*** has been selected for funding.

Each application was objectively reviewed, discussed, and scored by a peer review panel. Panel members evaluated applications according to the criteria published in the application packet. I am enclosing a copy of the comment sheets prepared by the peer reviewers. If you wish to discuss the peer review process, the reviewers' comments or any related matters pertaining to your application or NIDILRR's Field Initiated Program, please contact Theresa San Agustin, MD or Timothy Beatty at Theresa.SanAgustin@acl.hhs.gov; Timothy.Beatty@acl.hhs.gov.

Thank you for your interest in the FIP Grants. I look forward to your continued interest in NIDILRR grant programs.

Sincerely,

Kristi Hill, PhD
Director (Acting)
National Institute on Disability, Independent Living, and Rehabilitation Research

Enclosures

Applicant Final Panel Summary Report

Average Score: 94.20

Application Number: IFRE17000009
Application Name: Medical University of South Carolina
State: SC City: Charleston

Criteria Name(Max Score)

1. Importance of the problem (20 Points)
2. Design of research activities (50 Points)
3. Plan of evaluation (5 Points)
4. Project staff (15 Points)
5. Adequacy and accessibility of resources (10 Points)

TOTAL: 100

Strength:

The panel agreed on multiple strengths that were seen throughout all sections of the application. These strengths are listed in the individual reviews.

The need and target population have been clearly described.

The project's capability to further the purposes of the Act is presented.

Potential beneficial impact on the target population has been detailed.

A clear focus on feasibility and acceptability testing of a (new) intervention is established (Intervention Development).

Well informed lit review and theoretically sound logics are used.

Adequate sample size.

PHOENIX taskforce involvement is a strength.

A thorough plan of evaluation for periodic assessment is presented.

Several related preliminary studies have been performed by this team and that highlights the skills and strengths of the team.

Adequacy of overall resources was well established.

Accessibility of the built environment was presented.

Weaknesses:

While all panel members noted multiple strengths in the Design of Research Activities section, several panel members mentioned weaknesses. Most of these were related to the Phase II study; equivalence of the treatment and control group, how treatment dropouts will be handled, and how the treatment materials will be developed. Specific concerns are listed here: Treatment assignment will not be stratified by time since injury, or by injury level.

There is no clarity on what constitutes the completion of the study by a participant and how dropouts will be treated.

For people with a longer time from injury, the value of the strategies they have already developed is not discussed.

There seems to be very little details/description on the method to produce the videos and the potential barriers. It was unclear if the applicant recognizes the complicated nature of creating meaningful videos and has access to individuals or companies who have the expertise to do so.

One reviewer expected additional methods of providing feedback (such as focus groups).

One reviewer noted that equal opportunity does not equate to actively recruiting underrepresented groups.

Computer access and transportation access details were not clear.

Resources for development of the multimedia materials in alternate sources was not addressed.

Panel reviewers displayed different expectations for what was required regarding the accessibility of resources. Some felt the information presented was sufficient, while others were looking for additional details for the accessibility of materials, technology and dissemination.

General Feedback:

.

Technical Review Form : 1

Criteria Name(Max Score) : Actual Score

1. Importance of the problem (20 Points) : 20
2. Design of research activities (50 Points) : 48
3. Plan of evaluation (5 Points) : 5
4. Project staff (15 Points) : 13
5. Adequacy and accessibility of resources (10 Points) : 8

TOTAL: 94 / 100

Rationale

Scoring Criteria

Criterion 1: Importance of the problem

Strength:

Page: 1-5

Applicant clearly describes the need and target population (10 points).The proposed activities further the purposes of the Act and proposed project will have beneficial impact on the target population

Weakness:

None

General:

None

Criterion 2: Design of research activities

Strength:

Page: research activities

strong application. Provides a thorough review of the literature. Good inclusion of individuals with SCI in the development and implementation of research. Stage of research appropriately identified.

Weakness:

Page: 9-40

Unclear about what counts as completion of intervention and if you are going to be continuing to track individuals who stop engaging with a peer navigator.

General:

None

Criterion 3: Plan of evaluation

Strength:

Page: 40-43

Nice inclusion of workplan. Feedback loop to improve performance in place

Weakness:

None

General:

None

Criterion 4: Project staff

Strength:

None

Weakness:

Page: 47-48

Equal opportunity practices is not the same as adopting practices which outreach and engage individuals from traditionally underrepresented groups. This should be specifically addressed.

General:

None

Criterion 5: Adequacy and accessibility of resources

Strength:

None

Weakness:

Page: 48-50

The applicant does not list access to a media company in their resource section. Given the complexity of creating videos to teach skills and engage the learner, it seems that access to this expertise is needed. In addition accessibility should include accessibility of project materials and outputs as opposed to just accommodations that will be made.

Page: resources

The applicant provides only limited information about access to technology resources and support to convert program to info into a web format would be useful

General:

None

Technical Review Form : 2

Criteria Name(Max Score) : Actual Score

1. Importance of the problem (20 Points) : 19
2. Design of research activities (50 Points) : 50
3. Plan of evaluation (5 Points) : 3
4. Project staff (15 Points) : 15
5. Adequacy and accessibility of resources (10 Points) : 10

TOTAL: 97 / 100

Rationale

Scoring Criteria

Criterion 1: Importance of the problem

Strength:

Page: 1-9

The target population is well described. The importance of the problem is very well documented and shows a profound and wide ranging appreciation of the problem of self-management service models for people with SCI. The relationship to the act is good. The impact of peer-supported self management and telehealth has been provided adequately.

Weakness:

Page: 1-9

The impact of peer-supported self management and telehealth has been provided in terms of possibly better interventions with activities of quality of life even if more details could be provided such as financial savings, measures of performance, etc.

General:

None

Criterion 2: Design of research activities

Strength:

Page: 10-40

The research hypothesis concept is sound and there is no doubt about the importance of the proposed self-management and teleheath system for the overall quality of life of the targeted beneficiaries. Thus, the concept is described and illustrated effectively while the participating population is of sufficient size. The data analysis and collection methods are appropriate while the input of individuals with disabilities is used to shape the proposed research activities. The applicant identifies and justifies the stage of research being proposed and the research methods associated with the stage.

Weakness:

None

General:

None

Criterion 3: Plan of evaluation

Strength:

Page: 40-42

The appropriateness of monitoring and evaluation methods (e.g. meetings) have been demonstrated with sufficient precision for the proposed project cycle by following the agile methodology.

Weakness:

Page: 40-42

More details and more methods/protocols (e.g. focus groups) could be provided for the evaluation and the quality of the proposed work. Additionally, the method of providing feedback to the project staff as the project proceeded was not clear.

General:

None

Criterion 4: Project staff

Strength:

Page: 43-47

Staff is very well qualified and diverse.

Weakness:

None

General:

None

Criterion 5: Adequacy and accessibility of resources

Strength:

Page: 48-50

Adequate

Weakness:

None

General:

None

<u>Technical Review Form : 3</u>

Criteria Name(Max Score) : Actual Score

1. Importance of the problem (20 Points) : 20
2. Design of research activities (50 Points) : 45
3. Plan of evaluation (5 Points) : 5
4. Project staff (15 Points) : 15
5. Adequacy and accessibility of resources (10 Points) : 10

TOTAL: 95 / 100

Rationale

Scoring Criteria

Criterion 1: Importance of the problem

Strength:

<u>Page: 1-9</u>

This part has been very well written. Need and target populations are very well described. All important aspects have been covered. The use of peer mentors is a good idea. Use of a person with similar conditions can help. The study definitely furthers purposes of the act by generating new scientific knowledge on community-based self-management interventions that are designed to optimize the health, independence, community participation, and QOL of individuals with SCI. At the end of the study, the outcome will be improved QOL after SCI by increasing self-management capacity leading to an increase in community participation and prevention of medically serious secondary conditions.

Weakness:

None

General:

None

Criterion 2: Design of research activities

Strength:

None

Weakness:

Page: 9-40

Too much information from the previous study actually creates a few obstacles in the reading of this section, throughout the application. The inclusion criteria is very broad especially with respect to time since injury (p.30). People with an injury for many years have developed strategies to address some of their problems. So it is not clear what exact purpose the video will be serving. Qualitative data analysis needs to be better defined. it is unclear as to which approach is being used for the qualitative data analysis.

General:

None

Criterion 3: Plan of evaluation

Strength:

Page: 40-42

Regular meetings being held very 2 years and feedback is appropriately sought from the SCSCIA groups as well and incorporation of feedback.

Weakness:

None

General:

None

Criterion 4: Project staff

Strength:

Page: 42-48

The project staff members are adequately qualified to carry out the proposed activities.

Weakness:

None

General:

None

Criterion 5: Adequacy and accessibility of resources

Strength:

Page: 48-50

The applicant is committed to provide adequate facilities, equipment, and other resources, including administrative support, and laboratories. All information has been elegantly described. The facilities, equipment, and other resources are appropriately accessible to individuals with disabilities who may use the facilities, equipment, and other resources of the project. They have mentioned that all areas are accessible.

Weakness:

None

General:

None

Technical Review Form : 4

Criteria Name(Max Score) : Actual Score

1. Importance of the problem (20 Points) : 18
2. Design of research activities (50 Points) : 45
3. Plan of evaluation (5 Points) : 5
4. Project staff (15 Points) : 13
5. Adequacy and accessibility of resources (10 Points) : 8

TOTAL: 89 / 100

Rationale

Scoring Criteria

Criterion 1: Importance of the problem
Strength:

Page: not specific
The need and target population have been clearly described.

Weakness:

Page: not specific
Scalability of the project would be improved if telehealth was used not only for content delivery and communication, but also to help alleviate the information and cognitive load of the peer mentors, thereby increasing the potential impact of this project.

General:

None

Criterion 2: Design of research activities
Strength:

Page: not specific
A clear focus on feasibility and acceptability testing of a (new) intervention is established.

Weakness:

Page: not specific

There seems to be very little details/description on the method to produce the videos and the expected barriers. The value provided to people with already developed strategies to handle the secondary conditions being considered was unanswered.

General:

None

Criterion 3: Plan of evaluation

Strength:

Page: not specific

A very detailed plan of evaluation and feedback mechanism has been proposed.

Weakness:

None

General:

None

Criterion 4: Project staff

Strength:

Page: not specific

Several preliminary studies have been performed by this team in this area and shows the skills and strengths of the team.

Weakness:

Page: not specific

Equal opportunity does not equate to actively recruiting underrepresented groups.

General:

None

Criterion 5: Adequacy and accessibility of resources

Strength:

Page: not specific

An overall adequate amount of resources have been identified.

Weakness:

Page: not specific

Computer access and transportation access details were not clear. Resources for development of the multimedia materials was not addressed.

General:

None

Technical Review Form : 5

Criteria Name(Max Score) : Actual Score

1. Importance of the problem (20 Points) : 20
2. Design of research activities (50 Points) : 46
3. Plan of evaluation (5 Points) : 5
4. Project staff (15 Points) : 15
5. Adequacy and accessibility of resources (10 Points) : 10

TOTAL: 96 / 100

Rationale

Scoring Criteria

Criterion 1: Importance of the problem

Strength:

Page: 85

The application clearly describes the target population as individuals who have a spinal cord injury (SCI), and the needs of the target population. The link between the proposed activities and the Act is clearly stated. The proposed intervention will clearly be beneficial to individuals in the target population.

Weakness:

None

General:

None

Criterion 2: Design of research activities

Strength:

Page: 93

The application clearly describes and justifies the state of research as intervention development.

Page: 93

The two phase project is designed to obtain and utilize ample input from individuals with disabilities and other stakeholders.

Page: 93

In order to evaluate the feasibility of the outcome measures, the data sources are appropriate. The proposed data analyses are appropriate for the stage of research.

Page: 93

The application contains an informed literature review, and the research questions are appropriate and theoretically sound for the proposed research.

Page: 93

The applicants provide documentation for a large and diverse sample for enrollment.

Page: 93

The sampling frame and sample size are appropriate given the state of research.

Weakness:

Page: 93

The inclusion and exclusion criteria for Phase II do not specify or limit enrollment based on time since an individual experienced a spinal cord injury. This may be an important factor. The application proposes simple random assignment of 15 individuals each to active treatment or wait list control. There is no assurance that the two groups will be comparable on important factors such as severity of injury, or access to services.

General:

None

Criterion 3: Plan of evaluation

Strength:

Page: 124

The application contains a plan of evaluation with appropriate feedback loops for Phase II. Because Phase II is a feasibility study, this portion of the study has a very intensive plan of evaluation. The study team will meet every two weeks during Phase II.

Weakness:

None

General:

Page: 124

The application states "During year 1, the PTF will meet monthly to assist with development of the educational videos and provide feedback on the development and layout of the iTunes U course." Performance activities will include"(a) ongoing evaluation of progress through regular meetings, (b) adherence to the work plan, and (c) implementation of corrective measures. We will review the extent to which we are following timelines and implementing all proposed activities, evaluate the extent to which the proposed activities are being successful, and implement corrective action in the unexpected situation that one or more of the activities falls outside the intended goal. Evaluation will be ongoing throughout the grant."

Criterion 4: Project staff

Strength:

Page: biographical sketches

The key investigators have conducted a series of preliminary studies in this area and are well suited to conduct the proposed project.

Weakness:

None

General:

None

Criterion 5: Adequacy and accessibility of resources

Strength:

Page: 132

The application clearly describes resources that are adequate for the proposed project, and the accessibility of the site.

Weakness:

None

General:

None
